# Brain dynamics reflecting an intra-network brain state is associated with increased posttraumatic stress symptoms in the early aftermath of trauma

**DOI:** 10.21203/rs.3.rs-4004473/v1

**Published:** 2024-03-08

**Authors:** Mohammad Sendi, Zening Fu, Nathaniel Harnett, Sanne van Rooij, Victor Vergara, Diego Pizzagalli, Nikolaos Daskalakis, Stacey House, Francesca Beaudoin, Xinming An, Thomas Neylan, Gari Clifford, Tanja Jovanovic, Sarah Linnstaedt, Laura Germine, Kenneth Bollen, Scott Rauch, John Haran, Alan Storrow, Christopher Lewandowski, Paul Musey, Phyllis Hendry, Sophia Sheikh, Christopher Jones, Brittany Punches, Robert Swor, Nina Gentile, Vishnu Murty, Lauren Hudak, Jose Pascual, Mark Seamon, Erica Harris, Anna Chang, Claire Pearson, David Peak, Roland Merchant, Robert Domeier, Niels Rathlev, Brian O’Neil, Paulina Sergot, Leon Sanchez, Steven Bruce, John Sheridan, Steven Harte, Ronald Kessler, Karestan Koenen, Samuel McLean, Jennifer Stevens, Vince Calhoun, Kerry Ressler

**Affiliations:** Harvard Medical School/McLean Hospital; d Data Science (TReNDS), Georgia State University, Georgia Institute of Technology, Emory University; McLean Hospital; Emory University School of Medicine; Blue Halo; Harvard Medical School/McLean Hospital; McLean Hospital, Harvard Medical School; Washington University School of Medicine; The Alpert Medical School of Brown University, Rhode Island Hospital and The Miriam Hospital; University of North Carolina at Chapel Hill; San Francisco VA Healthcare System; University of California San Francisco; Emory University School of Medicine; Georgia Institute of Technology; Wayne State University School of Medicine; University of North Carolina at Chapel Hill; McLean Hospital; University of North Carolina Chapel Hill; McLean Hospital; University of Massachusetts Medical School; Vanderbilt University Medical Center; Indiana University School of Medicine; University of Florida College of Medicine -Jacksonville; Cooper Medical School of Rowan University; University of Cincinnati College of Medicine & University of Cincinnati College of Nursing; Beaumont Hospital; Temple University; Temple University; Emory University School of Medicine; Perelman School of Medicine at the University of Pennsylvania; University of Pennsylvania; Einstein Medical Center; Jefferson University Hospitals; Wayne State University; Ascension St. John Hospital; Massachusetts General Hospital; Brigham and Women’s Hospital; Saint Joseph Mercy Hospital; University of Massachusetts Medical School-Baystate; Wayne State University School of Medicine; Department of Emergency Medicine, McGovern Medical School at UTHealth; Brigham and Women’s Hospital; University of Missouri-St. Louis; Ohio State University College of Dentistry; University of Michigan; Harvard Medical School; Harvard School of Public Health; University of North Carolina; Emory University School of Medicine; Georgia Institute of Technology, Emory University and Georgia State University; McLean Hospital

## Abstract

This study examines the association between brain dynamic functional network connectivity (dFNC) and current/future posttraumatic stress (PTS) symptom severity, and the impact of sex on this relationship. By analyzing 275 participants’ dFNC data obtained ~2 weeks after trauma exposure, we noted that brain dynamics of an inter-network brain state link negatively with current (r=−0.179, *p*_*corrected*_= 0.021) and future (r=−0.166, *p*_*corrected*_= 0.029) PTS symptom severity. Also, dynamics of an intra-network brain state correlated with future symptom intensity (r = 0.192, *p*_*corrected*_ = 0.021). We additionally observed that the association between the network dynamics of the inter-network brain state with symptom severity is more pronounced in females (r=−0.244, *p*_*corrected*_ = 0.014). Our findings highlight a potential link between brain network dynamics in the aftermath of trauma with current and future PTSD outcomes, with a stronger protective effect of inter-network brain states against symptom severity in females, underscoring the importance of sex differences.

## Introduction

Post-traumatic stress disorder (PTSD) may develop in individuals who have experienced or witnessed a traumatic event, such as military warfare, sexual or physical assault, accidents, or natural disasters ^1^. Symptoms of PTSD include distressing thoughts, flashbacks, avoidance of reminders, changes in mood and cognition, and increased arousal, which can significantly impact an individuals’ life ^2^. Biological markers or biomarkers may be able to identify those who are more likely to develop PTSD following a traumatic incident ^3,4^. Early identification of such individuals might allow for prompt treatment and preventive measures, potentially minimizing the severity and duration of PTSD symptoms. Furthermore, these markers may help in the development of tailored treatment methods, the optimization of therapeutic treatments, and the long-term monitoring of therapy response ^5^.

In recent years, there has been a significant increase in the exploration and advancement of neuroimaging-based markers for identifying vulnerability to PTSD ^6,7^. This emerging field shows great potential in the rapid development of tools for early identification and intervention ^8^. Studies utilizing neuroimaging techniques have uncovered notable alterations in brain function among individuals with PTSD. These alterations are marked by atypical functional network connectivity (FNC) patterns, as observed in resting-state functional magnetic resonance imaging (fMRI) studies ^9–11^. Specifically, these patterns are seen in various brain regions, including the hippocampus ^12^, amygdala ^13^, visual network ^14^, and prefrontal cortex ^13^ in individuals with PTSD. This underscores the extensive influence of trauma on brain networks. Furthermore, several studies have successfully utilized resting-state fMRI functional connectivity to predict the severity of PTSD symptoms ^15–18^. Additionally, two recent studies revealed the ability to predict future symptom severity in participants with PTSD by analyzing resting-state fMRI data obtained after the trauma had occurred ^19,20^.

It has been assumed that brain FNC remains quasi-static or invariant over long periods of time, leading many previous studies to focus solely on static FNC (sFNC) while ignoring the brain dynamics during rest. However, challenging this assumption, a relatively new concept called dynamic FNC (dFNC) has emerged ^21–25^. A dynamic approach recognizes that FNC during the relatively short length of resting-state fMRI scans can exhibit temporal variations, thereby highlighting the importance of studying the dynamic aspects of FNC ^26^. Unlike sFNC, dFNC offers greater sensitivity in capturing the spontaneous adaptations that occur in response to various cognitive and mental conditions ^27^. By considering the spontaneously fluctuating nature of neural signals across different temporal scales, dFNC allows for a more sophisticated evaluation of brain activity ^28^.

Considering the dynamic nature of FNC in resting-state fMRI, several studies have explored dFNC in the context of PTSD in recent years ^29–32^. However, none of these studies have examined the capability of dFNC to predict future PTSD symptom severity. In addition, previous research indicates that women are two to three times more likely than men to develop PTSD ^33^. Despite this, there has been a notable absence of studies that examine the potential effects of sex on the relationship between dFNC features and the severity of current or future PTSD symptoms.

In the present study, we aim to build upon previous research on dFNC in the context of PTSD. Specifically, we investigated the predictive capability of dFNC features for future PTSD symptom severity. Additionally, we explored the potential effects of sex on the association between dFNC features and both current and future symptom severity. As past studies have demonstrated, biological sex is not the primary determinant of the various neurophenotypes associated with adverse post-traumatic outcomes. Instead, a range of other factors such as low socioeconomic status or SES, including income ^34,35^, housing quality^36^, and broader socioeconomic conditions, area deprivation index or ADI^37^ also significantly influence the risk and severity of PTSD. To address the contribution of these factors, we also included them as covariates in our analysis.

To accomplish these goals, we utilized the dataset from the Advancing Understanding of Recovery after Trauma (AURORA) project ^38^. In the AURORA study, understanding whether dFNC features derived from resting-state fMRI early after a trauma can predict future PTSD symptom severity is crucial. This is especially true since neuroimaging was conducted approximately two weeks after the traumatic event, at a time when acute stress disorder may be assessed, but before the diagnosis of PTSD can be made. This timing allows us to investigate the potential of dFNC features as early biomarkers for PTSD and evaluate their predictive capability for the severity of PTSD symptoms at a later stage.

## Results

### Participants

**Figure 1:**
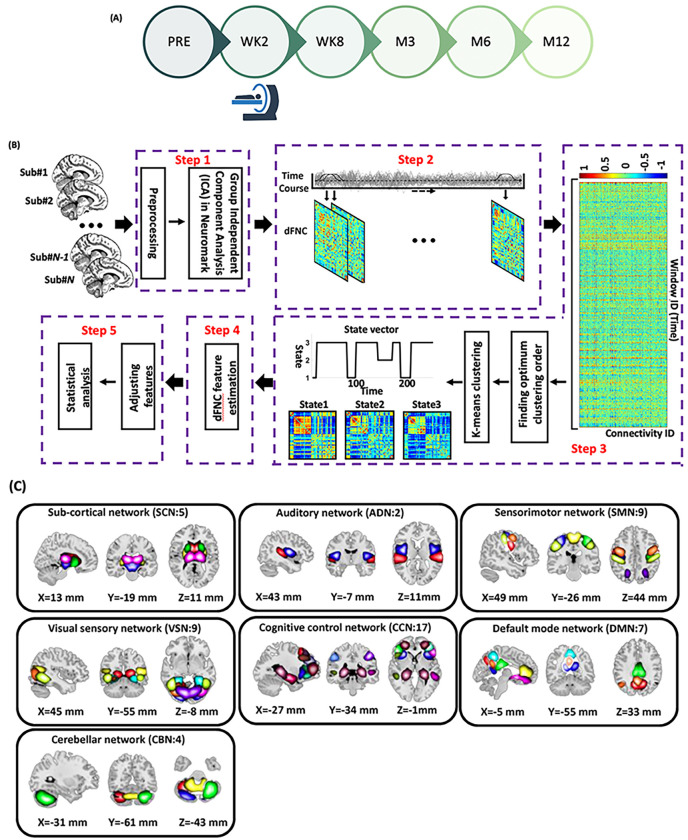
Data collection procedure and analytic pipeline: **A)** The PTSD Checklist for DSM-5 (PCL-5) was utilized to evaluate PTSD symptoms at various time points, encompassing pre-trauma (PRE), week 2 (WK2), week 8 (WK8), month 3 (M3), month 6 (M6), and month 12 (M12). During the study visit at WK2 a subgroup of participants underwent MRI scans, either in the morning or the afternoon. **B)** Dynamic functional network connectivity (dFNC) analytic pipeline: Step 1: Initially, the time-course signal of 53 intrinsic connectivity networks (ICNs) was identified through group-ICA in the Neuromak template. Step 2: Subsequently, the identified 53 ICNs were subjected to a taper sliding window segmentation to calculate FNC. Each subject yielded 201 FNCs, each with a size of 53 × 53. Additionally, static FNC was computed for the entire recording duration. Step 3: To cluster the FNCs into three distinct groups, the FNC matrices were vectorized and concatenated, followed by the utilization of k-means clustering with correlation as the distance metric. Step 4: From the state vector, occupancy rate (OCR) was computed for each subject, resulting in a total of three OCR features for each subject. Step 5: In order to investigate the relationship between OCRs with the PTSD clinical measure (i.e, PCL-5), we used GLM to compute the associations, taking into account factors such as age, sex, years of education, scanning site, income, marital status, employment status, and percentile ADI. The resulting t-statistics from this analysis were then converted to correlation (r) values. **C)** We utilized the NeuroMark pipeline to extract robust intrinsic connectivity networks (ICNs), totaling 53 components, which demonstrate consistent replication across independent datasets. These 53 distinct components were initially identified through group-ICA analysis using the NeuroMark template. These components were subsequently categorized into seven distinct networks, which include the subcortical network (SCN), auditory network (AND), visual sensory network (VSN), sensorimotor network (SMN), cognitive control network (CCN), default mode network (DMN), and cerebellar network (CBN).

Data for the current analyses were collected as part of the multisite emergency department (ED) AURORA study. The AURORA study represents a significant research effort aimed at enhancing our understanding, prevention, and recovery strategies for individuals who have undergone a traumatic event. In AURORA study, trauma-exposed civilians brought to one of 29 participating EDs across the United States were recruited for this large, longitudinal study (details in^38^). This study involved more than 4000 participants from the AURORA project, who provided clinical data at various intervals: 2 weeks (WK2), 4 weeks (WK4), 3 months (M3), 6 months (M6), and 12 months (M12) as illustrated in Fig. 1A. Additionally, neuroimaging data from ~ 400 participants were collected at WK2 from five different scanning locations, which include Atlanta (Georgia), Belmont (Massachusetts), Philadelphia (Pennsylvania), St. Louis (Missouri), and Detroit (Michigan). The recruitment for this study took place between September 2017 and December 2020 (Final freeze 4 Psychometric release). We excluded those with low-quality resting-state fMRI and missing clinical information at the imaging acquisition date. This process resulted in 275 participants (181 females) being included in this analysis. Table 1 summarizes the demographic characteristics of the participants included in this study.

### Three distinct dFNC states were identified

**Figure 2:**
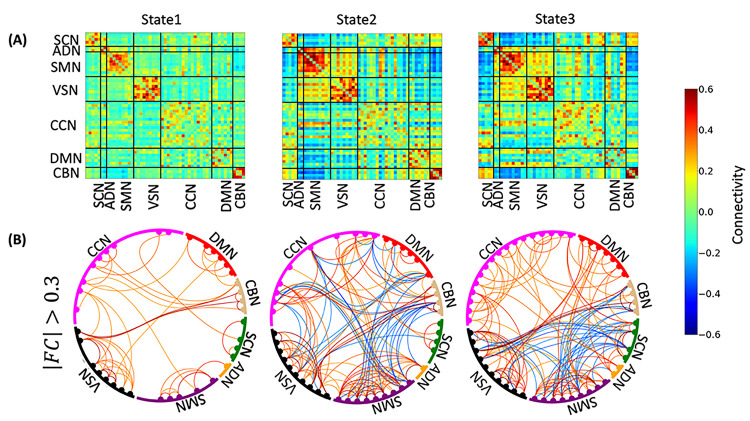
Three dynamic functional connectivity states identified in AURORA dataset. **A)** Three dynamic functional connectivity (dFNC) state identified using k-means clustering method. **B)** To enhance clarity, the dFNC state displayed by removing connectivities with values between −0.3 and 0.3. States 2 and 3 exhibit stronger positive connectivity among sensory networks (visual, auditory, and sensorimotor). State 1, on the other hand, shows more disconnections within these networks. State 3 demonstrates increased within-CCN connectivity and enhanced connectivity between the DMN and sensory networks compared to state 2. State 3 also exhibits greater connectivity between the CBN and SCN compared to the other two states. Overall, our analysis identifies states 2 and 3 as inter-network brain state while state 1 appears to be an intera-network brain state based on connectivity patterns. The color bar indicates the strength of the connectivity. SCN: Subcortical network; AND: auditory network; SMN: sensorimotor network; VSN: visual network; CCN: cognitive control network; DMN: default-mode network; and CBN: cerebellar network.

After calculating the dFNC of each participant, we grouped their dFNC into three different dynamic network connectivity states (Fig. 1B). Figure 2 presents an overview of the identified states. Each state represents 1378 connectivity measures among seven networks across the entire brain. These networks included subcortical network (SCN), auditory network (ADN), sensorimotor network (SMN), visual network (VSN), cognitive control network (CCN), default-mode network (DMN), and cerebellar network (CBN). The top panel highlights three distinct dFNC states, while the bottom panel shows the data with connectivities between − 0.3 and 0.3 removed for clarity. State 2 and state 3 exhibit a stronger positive connectivity among sensory networks, including visual, auditory, and sensorimotor networks. Conversely, in state 1, we observed more disconnections among these networks. We observed an increase in within-CCN connectivity and enhanced connectivity between the DMN and sensory networks in state 3. Additionally, we noted a greater connectivity between the CBN and SCN in state 3 compared to the other two states. Overall, our analysis suggests that state 2 and state 3 exhibit characteristics of inter-network states, evidenced by the increased connectivity across the seven networks. In contrast, state 1 is indicative of an intra-network state, as it demonstrates predominantly within-network connectivity patterns.

### Dynamic FNC occupancy rates link with PCL-5 scores.

**Figure 3:**
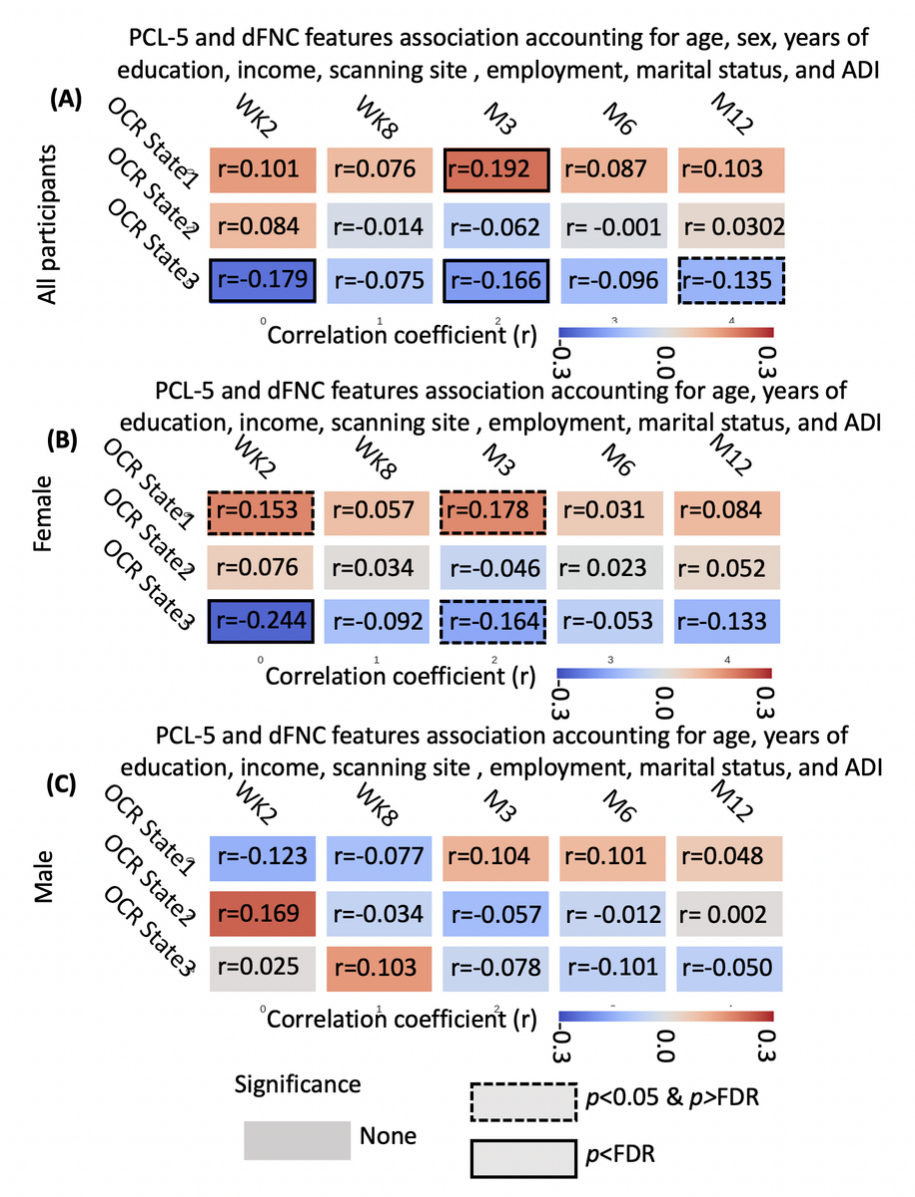
Dynamic functional network connectivity occupancy rates (OCRs) link with PCL-5 . **A)** The correlation between OCRs estimated from WK2 resting-state fMRI recording and PCL-5 if different time point for the whole population. Occupancy rate (OCR) of state 1 and state 3 link with WK2 and M3 PCL-5. **B)** The correlation between OCRs estimated from WK2 resting-state fMRI recording and PCL-5 if different time point for the female group. Occupancy rate (OCR) of state 1 and state 3 link with WK2 and M3 PCL-5. **C)** The correlation between OCRs estimated from WK2 resting-state fMRI recording and PCL-5 in different time point for the male group. No association has been observed. WK2: week 2 after trauma, WK8: week 8 after trauma, M3: month 3 after trauma, M6: month 6 after trauma, and M12: month 12 after trauma. Correlations with associated p-values < 0.05 are highlighted with a dashed line box, while correlations with associated False Discovery Rate (FDR) p-values < 0.05 are emphasized with a solid line box. The color bar represents the strength of correlation.

By utilizing the three identified brain states for the entire group and the state vector, estimated for each individual, which represents the state of the brain network at any given time point, we calculated three occupancy rates (OCRs) for each participant. The OCR of each state represents the proportion of time each participant spends in that state (see Method Section and Supplementary Fig. 1). Figure 3A shows the correlation between OCRs and PCL-5 scores at various time points. The associations were computed using General Linear Model (GLM) accounting for age, sex, years of education, scanning site, income, marital status, employment status, and percentile ADI, and the resulting t-statistics were transformed to correlation (r). A positive significant association was found between the OCR of state 1 and the PCL-5 scores at M3 (r = 0.192, β = 0.0039, SE = 0.0012, 95% CI: 0.0015 ~ 0.0062, *p*_*corrected*_ = 0.021, N = 226 after excluding sample with missing scores, see Table 1). These results indicate that the participants with higher PTSD symptom severity spend more time in state 1, which is indicative of an intera-network brain state.

We observed significant negative association between the OCR of state 3 and the PCL-5 scores at WK2 (r=−0.179, β= −0.0029, SE = 0.0009, 95% CI: −0.0048~−0.0010, *p*_*corrected*_= 0.021, N = 275). We also found a negative correlation between state 3 OCR and PCL-5 of M3 (r=−0.166, β=−0.0030, SE = 0.0011,95% CI: −0.0052~−0.0008, *p*_*corrected*_ = 0.029, N = 226). This indicates that individuals with higher PCL-5 scores spent less time in state 3, which is indicative of an inter-network brain state. Overall, our findings highlight the relationships between the OCR and PCL-5 scores, suggesting potential connections between dynamic functional network connectivity and symptoms of PTSD at different time points.

### Sex modulates the relationship between OCRs and PCL-5 scores.

To examine the influence of sex on the relationship between OCRs and PCL-5 scores, we conducted GLM analyses for males (N = 94) and females (N = 181), separately. In these analyses, we included age, years of education, scanning site, income, marital status, employment status, and ADI as covariates. The correlation results between OCRs and PCL-5 scores for females and males are presented in Fig. 3B and Fig. 3C, respectively. While no significant association was found between OCRs and PCL-5 scores in the male group, we did observe significant associations between the OCR of state 1 and state 3 with PCL-5 scores at WK2 and M3. Notably, only the association between OCR of state 3 and PCL-5 at WK2 remained significant after applying FDR correction (r=−0.244, β= −0.0030, SE = 0.0011, 95% CI: −0.0048~−0.0010, *p*_*corrected*_ = 0.014, N = 181). We also observed a positive link between state 1 OCR and WK2 PCL-5 (r = 0.153, β = 0.0027, SE = 0.0013, 95% CI: −0.0003 ~ 0.0040, *p*_*uncorrected*_ = 0.0402, N = 181). Additionally, OCR of state 1 showed a positive link with M3 PCL-5 (r = 0.178, β = 0.0034, SE = 0.0014, 95% CI: 0.0015 ~ 0.0062, *p*_*uncorrected*_ = 0.0167, N = 154) and OCR of state 3 showed a negative link with M3 PCL-5 (r=−0.164, β=−0.0028, SE = 0.0012, 95% CI: −0.0052~−0.0008, *p*_*uncorrected*_ = 0.0273, N = 154). However, none of them were significant after FDR correction.

To verify that the strong correlation in females is not due to their larger sample size compared to males, we tested the correlations between state 3 OCR and WK2 PCL-5 scores in both groups. Using Fisher’s z-transformation and calculating standard errors, we found a significant difference in the correlations between females and males (|Z-test statistic| = 1.734, p = 0.041), suggesting that the relationship between OCRs and PCL-5 scores at WK2 differs significantly between sexes.

### Both posttraumatic stress (PTS) and non-PTS group generate similar dFNC states

We categorized participants into posttraumatic stress or PTS (N = 124) and non-PTS (N = 151) groups based on their WK 2 PCL-5 scores, with a cutoff point of 31. Those scoring above 31 were classified as PTS, while those below were considered non-PTS ^39^. We used the term PTS instead of PTSD because the classification was based on PCL-5 scores at the time of imaging (i.e., WK2), before an official PTSD diagnosis till WK8. We then examined state pattern differences between the two groups by performing separate k-means clustering analyses on their dFNC data.

**Figure 4:**
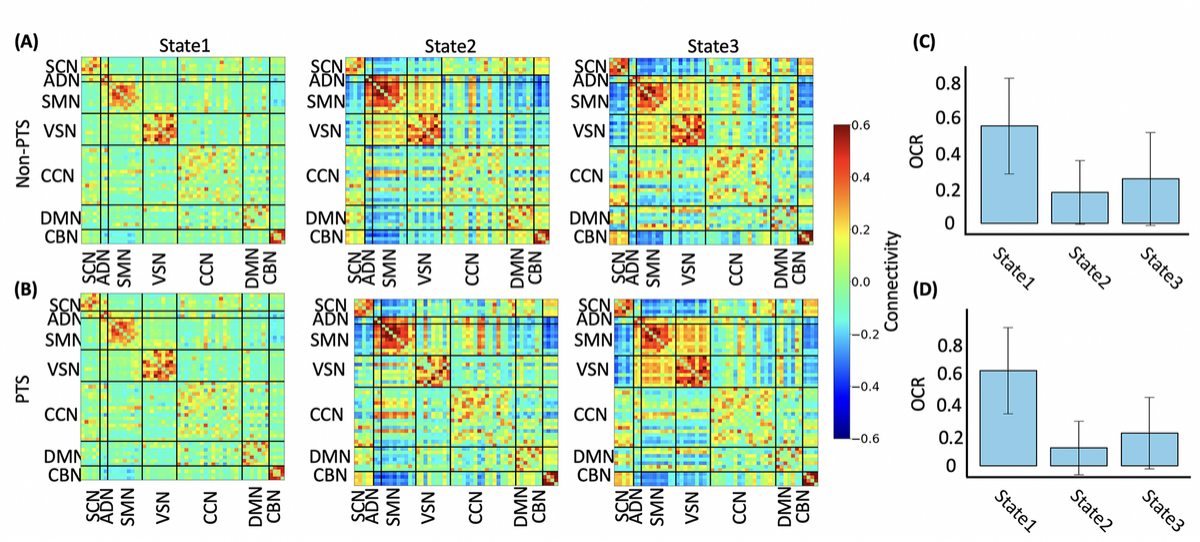
Both non-PTS and PTS group generate similar dynamic functional connectivity (dFNC) state. **A)** the dFNC states identified only in non-PTS group. **B)** the dFNC states identified only in PTS group. The color bar indicates the strength of the connectivity. SCN: Subcortical network; AND: auditory network; SMN: sensorimotor network; VSN: visual network; CCN: cognitive control network; DMN: default-mode network; and CBN: cerebellar network.

Figure 4 demonstrates a notable similarity in brain states between the PTS and non-PTS groups, as anticipated. We quantified the similarity by calculating the Pearson correlation coefficient between corresponding states’ FNC. The correlations between state 1 of the non-PTS group and state 1 of the PTS group, state 2 of the non-PTS group and state 2 of the PTS group, and state 3 of the non-PTS group and state 3 of the PTS group were 0.9632 (N = 1378, where N is number of connections, *p* ~ 0), 0.9880 (N = 1378, p ~ 0), and 0.8938 (N = 1378, *p* ~ 0), respectively (see Fig. 4A and 4B). The p-value, displayed as zero in MATLAB, indicates a very small value, suggesting strong statistical significance and reinforcing the robustness of our findings. Comparing the OCR of states in the non-PTS and PTS groups, we found a consistent pattern: state 1 consistently showed the highest OCR, while state 2 exhibited the lowest OCR in both groups (Fig. 4C and 4D). These results suggest a consistent OCR pattern across states in both groups, indicating a high degree of similarity in identified brain states between the non-PTS and PTS groups. Additionally, our findings that individuals with PTS tend to spend more time in state 1 compared to those without PTS corroborate our main finding that have established a connection between the heightened OCR of this state and PCL-5, hinting at the potential clinical relevance of this brain state in PTS.

## Discussion

Our current research aimed to investigate the significance of temporal changes in brain connectivity, measured by dynamic functional network connectivity (dFNC), in indicating the presence and severity of PTSD symptoms. Additionally, we examined the influence of sex-specific differences on the predictive ability of these connectivity measures. Our results indicate that the amount of time spent in an inter-network brain state serves as a protective factor against PTSD, whereas time spent in an intera-network brain state is linked to a higher PTSD symptom severity. Furthermore, we observed that the negative association between the duration spent in an inter-network brain state and PCL-5 is more pronounced in the female group.

Dynamic FNC offers an enhanced predictive power compared to static FNC (sFNC), supplying an additional layer of information about the severity of symptoms in brain disorders over time, a level of detail not attainable by its static counterparts ^40–42^. For instance, a recent study demonstrated that a classification model relying on dFNC features surpassed the performance of other classification models in patients diagnosed with multiple sclerosis ^42^. In another study involving participants with PTSD, the temporal variability, as captured by dFNC, demonstrated a higher classification accuracy than the model obtained only by sFNC features ^41^. Our study demonstrates that dFNC features associate not only with current traumatic stress symptoms, but predict future symptoms of PTSD and may reveal important sex differences.

In our study sample, comprising participants exposed to traumatic events, we analyzed dFNC and differentiated three distinct brain network states. Two out of the three states (i.e., state 2&3) exhibited a higher degree of integration in the sensory network, while state 1 demonstrated a more disconnected sensory network. State 3 manifested the strongest connectivity within the CCN, within the CBN, and between the CBN and the SCN. Moreover, we found that state 1 was characterized by intera-network connectivity, while the other two states exhibited inter-network connections with both strong negative and positive connectivity among brain networks. These observations collectively highlight that brain networks display substantial dynamism, a characteristic they maintain even without the presence of external stimuli as has been observed in other brain disorders ^21–25,29,40^. Additionally, we investigated whether the dynamics of brain networks in participants with PTS differed from those in the non-PTS group. Upon separately analyzing data from both groups of participants, we observed that each group generated similar dFNC states, as expected and observed in other disorders^43^. This suggests that the dynamic nature of brain networks persists irrespective of PTS, highlighting the potential complexities and resilience of the brain’s network dynamics in the face of trauma and related disorders.

A prior study, employing the same population as the current research, demonstrated that the static functional connectivity between the left dorsolateral prefrontal cortex (DLPFC) and the arousal network (AN), as well as between the right inferior temporal gyrus (ITG) and the default mode network (DMN), could predict both WK2 and M3 PCL-5 scores ^20^. In the current study, we found that the whole-brain OCRs estimated from dFNC predict the PCL-5 at the time of neuroimaging data collection (referred to as WK2), as well as the PCL-5 scores 10 weeks post-data collection (referred to as month 3 or M3). Our new analyses contribute to a deeper understanding of the neurobiological mechanisms underlying PTSD by looking at brain network dynamics.

Specifically, we found that participants with higher PCL-5 scores tend to spend more time in an intera-network brain state, referred to as state 1. Importantly, the amount of time spent in this state was found to predict future symptom severity at M3 (Fig. 3A). State 1 is characterized by reduced connectivity among sensory networks, including visual, auditory, and sensory motor networks. Furthermore, our results confirmed that spending more time in an inter-network brain state (state 3) is negatively correlated with PCL-5 scores at WK2 and M3 (Fig. 3A). State 3 is characterized by increased connectivity among sensory networks, suggesting enhanced information exchange and integration between these networks. Previous studies have consistently reported impairments in visual processing, as well as auditory processing, in individuals with PTSD ^44,45^. Multiple neuroimaging studies have demonstrated alterations in the functioning of the visual, auditory, and motor cortex among participants with PTSD ^45–47^. Notably, abnormal activation in the visual cortex during picture viewing tasks has been observed in these individuals ^45^. Furthermore, significant alterations in visual processing have been identified within the ventral visual stream, which is responsible for processing object properties ^45^. This suggests that PTSD affects the specific components of the visual system involved in object recognition and perception, as previous findings highlight a role for structural integrity of the ventral visual stream in the development of PTSD ^48,49^. Our current findings, in conjunction with previous reports of subtle deficits in sensory networks, particularly the visual sensory system in PTSD, provide compelling evidence that disruptions in information integration among sensory networks are closely linked to the severity of PTSD symptoms^48–51^. Enhancing the connectivity and integration within these networks could potentially serve as a therapeutic target for mitigating symptom severity and improving outcomes in individuals with PTSD ^52^.

In addition to the sensory networks, our findings reveal that state 1 is characterized by relatively lower within-CBN connectivity and between CBN and SCN connectivity (i.e., CBN/SCN) compared to the other two states. This observation aligns with previous structural neuroimaging studies that have reported reduced cerebellar volumes in individuals with PTSD ^53,54^. Furthermore, functional neuroimaging studies have provided corresponding evidence by demonstrating alterations in neural activity and functional connectivity of the cerebellum in PTSD ^55^. Our new finding, that participants with higher PCL-5 scores preferentially spent more time in the state characterized by lower CBN, adds another layer of information to the understanding of temporal network patterns associated with CBN in PTSD. This suggests that alterations in cerebellar connectivity patterns may play a role in modulating symptom severity and could serve as potential markers for the disorder.

In the subsequent analysis, we investigated the influence of sex on the relationship between brain network dynamics and symptom severity. We observed that the association between OCRs, and PCL-5 scores was more prominent in females. Specifically, the correlation between state 3 OCR and WK2 PCL-5 was statistically significant within the female group, and the strength of this correlation was notably higher among females compared to males (Fig. 3B and C). It is worth noting that previous studies have extensively explored the role of sex in the development of PTSD, with emerging evidence suggesting differences in symptomatology and underlying neurobiology between males and females ^33,56–60^. In line with these findings, our results further support the notion that the identified dFNC biomarkers, particularly when correlating with symptom severity, are stronger in females; this could potentially reflect the higher prevalence of PTS/PTSD in this demographic.

Recent large-scale genomic studies show that women of European and African ancestry may have higher heritability for PTSD than men, suggesting that genetic factors may also play a significant role in the disorder’s development, particularly in interaction with sex differences ^61,62^. However, it’s important to note that biological sex is not the primary determinant of the various neurophenotypes associated with adverse post-traumatic outcomes; other factors such as low socioeconomic status also play a significant role ^34,35^. To avoid a narrow focus on sex alone, our analysis took into consideration all available socioeconomic and demographic factors from the dataset. This approach allowed us to conduct a comprehensive analysis of the connection between OCRs and PTSD symptom severity, specifically considering the sex effect. Additionally, women’s risk for PTSD is partially determined by the fact that they experience sexual traumas more frequently. For example, a study shows that women exhibit almost twice the PTSD symptoms in sexual assault survivors^63^. However, in the AURORA dataset, the type of trauma does not play a major role in driving sex differences. The traumas are primarily motor vehicle collisions (MVCs) for both women and men, yet sex differences in dFNC link with PTSD sympthom severity are still observed.

Several limitations should be acknowledged while interpreting the present findings. The overall sample size was relatively modest, and the sample sizes amongst the comparison groups (male vs. female) were not the same. Furthermore, participants who completed all scans and had more complete datasets may differ from those who did not complete all scans, making it unclear if the results apply to dropouts who may be at higher risk for PTSD after trauma. In this study, we examined dFNC in individuals with PTS and a non-PTS group, both of whom were exposed to trauma. To gain a comprehensive understanding, further research is required to directly compare the dFNC features among the PTSD group, a group of healthy individuals exposed to a traumatic event, and a group of healthy individuals who have not undergone any traumatic experiences. However, we assume that healthy individuals exposed to trauma could serve as a more suitable control group for those with PTSD, facilitating our understanding of the underlying neural processes of PTSD. Additionally, in this study, we investigated the relationship between dFNC features and the severity of PTSD symptoms at various time points. However, to enhance our understanding, future research should compare dFNC features among groups exhibiting different PTSD trajectories during a one-year assessment. In our study, we utilized the initial neuroimaging data available from the AURORA study, which was collected two weeks post-trauma, before any PTSD diagnosis at week 8. Given that the AURORA study also gathered neuroimaging data at six months post-trauma, future research would benefit from examining the dFNC patterns using the resting-state fMRI data from this later time point. Such analysis could yield more profound insights into the evolving brain dynamics associated with PTSD.

## Conclusions

In summary, our investigation into the dFNC of civilians recently exposed to trauma revealed distinct patterns in brain network dynamics. Our findings indicate that the duration participants spent in certain brain network states can forecast both their current and subsequent PCL-5 scores. Specifically, we identified that spending time in an intra-network brain state is associated with higher PCL-5 scores, while engagement in an inter-network brain state correlates with lower PCL-5 scores. Furthermore, our analysis highlighted the role of multiple brain networks encompassing the visual, auditory, sensory-motor, and cerebellar networks, in PTSD. We also observed a stronger association between brain dynamics and PCL-5 scores in females compared to the male group. By incorporating sex-specific disparities, tailoring interventions and treatment strategies accordingly, we can potentially develop more effective and personalized approaches for PTSD.

## Methods

### Study population

The participants in this study are from the Advancing Understanding of Recovery after Trauma project (AURORA) (Freeze 4.0 dataset). AURORA is a multisite longitudinal study in which participants are enrolled within 72 hours of trauma exposure ^38^. In this study, the participants who experienced incidents like a car accident, a high fall (> 10 feet), a physical assault, sexual violence, or mass casualty incident were considered to have experienced trauma. The inclusion criteria include: 1) aged between 18 and 65 years old, 2) being alert and oriented at the Emergency Department (ED), 3) having the ability to speak and write English fluently, 4) having no cognitive impairment, 5) having the ability to use the smartphone for > 1-year post-enrollment. Exclusion criteria included solid organ damage, severe bleeding, a requirement for a chest tube, and the likelihood of being admitted for longer than 72 hours. A subset of participants underwent MRI either in the morning or the afternoon of the study visit, which occurred approximately two weeks after the traumatic event (i.e., WK2). After preprocessing and quality check, N = 275 participants’ data were used in our study.

### Clinical measures

The PTSD Checklist for DSM-5 (PCL-5) was administered to assess PTSD symptoms at multiple time points, including pre-trauma (PRE), week 2 (WK2), week 8 (WK8), month 3 (M3), month 6 (M6), and month 12 (M12), as depicted in Fig. 1A. It is important to emphasize that different time frames were considered for each of the time points: the 2-week (WK2) assessment reflected symptoms experienced over the past two weeks, while assessments from week 8 (WK8) onwards considered symptoms over the past 30 days. This longitudinal assessment allows for a comprehensive understanding of the participants’ PTSD symptomatology throughout the study duration. Table 1 summarizes the demographic and clinical characteristics of the participants included in this study. Additionally, to distinguish individuals with posttraumatic stress (PTS) from those without PTS in WK2 of the study, we employ a threshold for the PCL-5 at 31. Participants with a PCL-5 score greater than 31 are classified as having PTS, while those with a score less than 31 are considered non-PTS ^39^. It is important to note that we refer to this group as having PTS and not PTSD, as the PTSD diagnosis was made in W8, while we used the WK2 PCL-5 scores to identify these two groups.

### Imaging acquisition protocol

Participants underwent a thorough screening process before undergoing scanning, which involved checking for any contraindications to magnetic resonance imaging (MRI) or other exclusion criteria. For female participants and those who could potentially be pregnant, a pregnancy test was administered prior to entering the MRI environment. MRI scans were conducted using 3T Siemens scanners at each site. While the scan sequences remained largely consistent across imaging sites, some variations in sequence parameters were present due to differences in hardware. The imaging protocol for each site is outlined in Supplementary Table 1. The resting-state imaging procedure lasted approximately 9 minutes, during which participants were instructed to keep their eyes open. They were asked to focus on the white cross displayed at the center of the screen and maintain a state of stillness throughout the imaging session ^20^.

### Preprocessing

We corrected the differences in image acquisition times between slices using the statistical parametric mapping (SPM12 @ https://www.fil.ion.ucl.ac.uk/spm/) default slice timing routines. The slice acquired in the middle of the sequence was chosen as the reference slice. The subject’s head movement was then corrected using a rigid body, and 3-dimensional brain translations and 3-dimensional rotations were estimated. Next, the imaging data were resampled to 3 × 3 × 3 mm^3^. and spatially normalized to the Montreal Neurological Institute (MNI) space using the echo-planar imaging (EPI) template and the SPM toolbox’s default bounding box. The fMRI images were then smoothed using a Gaussian kernel with a full width at half maximum (FWHM) of 6 mm (Step1 in Fig. 1B). It should be emphasized that while participants in this study have also been featured in other AURORA analyses and resting-state studies^20,64^, the current analyses are distinct. Additionally, the preprocessing approach diverges from the standard protocols commonly employed in AURORA research in order to align with methodologies used in our other work. A similar preprocessing approach has been employed in several of our previous studies^23–25,43,65^.

### Extracting independent components using Neuromark

We applied a hybrid Neuromark framework to extract the meaningful networks for each subject. The Neuromark framework is based on the Neuromark template derived from two large datasets including the human connectome project (HCP: https://www.humanconnectome.org/study/hcp-young-adult/document/1200-subjects-data-release, 823 subjects after the subject selection) and genomics superstruct project (GSP: https://dataverse.harvard.edu/dataverse/GSP, 1005 subjects after the subject selection). This framework has been successfully implemented to many studies with a wide range of brain imaging markers identified across different brain diseases ^23–25,43,65^. Details of the construction of the templates can be found in our previous Neuromark paper ^66^.

The Neuromark template consists 53 independent components (ICs), which were grouped into seven functional networks based on anatomic and functional prior knowledge (Fig. 1C). These networks included subcortical network (SCN), auditory network (ADN), sensorimotor network (SMN), visual network (VSN), cognitive control network (CCN), default-mode network (DMN), and cerebellar network (CBN) (Step2 in Fig. 1B) ^67^. All 53 ICs and their coordination are shown in Supplementary Table 2. We used these priors (i.e., the Neuromark_fMRI_1.0 template, available in GIFT @ http://trendscenter.org/software/gift and on the TReNDS website @ http://trendscenter.org/data) to run a fully automated ICA analysis in GIFT ^68^. We further: 1) detrended linear, quadratic, and cubic trends, 2) conducted multiple regression on the six realignment parameters and their temporal derivatives, 3) despiked detected outliers, and 4) applied a low-pass filter (cut-off frequency at 0.15Hz) to remove noise and artifacts.

### Dynamic and static functional network connectivity estimation

The dFNC of the whole brain was estimated via a sliding window approach, as shown in Fig. 2B (Step 3). We used a tapered window obtained by convolving a rectangle (window size = 20 TRs = 47.2 s) with a Gaussian (σ = 3) to localize the dataset at each time point. Prior research revealed that a window size between 30 and 60 s is a suitable option for capturing dFNC variation ^69^. Thus, we assumed that a window size of 47.2 s is a reasonable choice. Next, within each window, we calculated the Pearson correlation between any pairs of ICs. We then concatenated the dFNCs of each participant to form a (C × C × T) array (where C = 53 denotes the number of ICs and T = 210), which represented the changes in brain connectivity between ICs as a function of time ^67^.

### Dynamic functional network connectivity clustering

We next concatenated the dFNC of all subjects, as shown in Step 4 of Fig. 1B, and applied the k-means clustering algorithm to the dFNC windows to partition the data into sets of distinct clusters representing transient connectivity “states” ^70,71^. The optimal number of cluster order was estimated using the elbow criterion based on the ratio of within to between cluster distances. By sweeping the k-value from 2 to 9, we found that the optimal number of clusters was 3 ^24^. We used Euclidian distance as a distance metric in this k-means clustering algorithm with 1000 iterations (Step 4 in Fig. 1B). The k-means clustering analysis yielded three distinct states across all 275 participants and a state vector for each individual.

The state vector reflects the temporal changes in whole-brain FNC. Subsequently, we determined the occupancy rate (OCR) for each participant, which is the proportion of time spent in each state. To compute the OCR for state *i* for a participant, we counted the number of windows in state *i* attributed to that participant and divided this by 210 (the total number of windows). Thus, we obtained three OCR values for each individual, corresponding to the three states. (Step 5 in Fig. 1B). Two representative state vectors of PTSD and non-PTSD individuals and their associated OCR for each state are shown in Supplementary Fig. 1.

### Statistical analysis

We employed a General Linear Model (GLM) to explore the association between OCRs and PCL-5 scores using data from all participants (N = 275). Our analysis included covariates such as age, sex at birth, years of education, income, employment status, marital status, scanning site, and percentile ADI. We constructed individual models for each OCR and time point, resulting in a total of 15 models derived from the combination of 3 predictors and 5 time points. Additionally, we developed 15 models for each sex group of males (N = 94) and females (N = 181). In the context of sex-stratified analyses, sex itself was excluded as a covariate, and the analysis was run separately for each sex group. Therefore, we had 15 models for the whole group analysis, 15 models for the female group analysis, and 15 models for the male group analysis. A Benjamini-Hochberg false discovery rate correction was applied to account for the 15 significance tests corresponding to the correlations of each analysis. In this study, all data analysis and statistical computations were conducted using MATLAB software (MathWorks, Natick, MA, USA) version R2022a.

## Figures and Tables

**Table 1:** Participant demographics and clinical information

Characteristics	Mean (SD) or N (%)
*Demographic characteristics*	
**Age**	34.55(12.78)
**Sex assigned at birth, male/female**	94 (34.18%)/181(65.82%)
* **Race/ethnicity**	
**Hispanic**	42 (15.27%)
**White**	85 (30.91%)
**Black**	131 (47.64%)
**Others**	15 (5.45%)
**Missing**	2 (0.73%)
**Years of education**	15.16(2.31)
**Income level**	
**<$19,000**	74 (25.96%)
**$19,001-$35,000**	85 (30.91%)
**$35,001-$50,000**	40 (14.55%)
**$50,001-$75,000**	30 (10.91%)
**$75,001-$100,000**	17 (6.18%)
**>-$100,000**	20 (7.27%)
**Missing**	9 (3.27)
*Clinical characteristics*	
**PCL-5 score**	
**WK2 (N = 275)**	30.12 (17.58)
**WK8 (N = 243)**	26.60 (17.30)
**M3 (N = 226)**	23.53 (17.40)
**M6 (N = 208)**	21.00 (17.33)
**M12 (N = 176)**	20.33 (17.93)

* Self-reported . SD: standard deviation, BMI: body mass index, WK2: 2 weeks after trauma, WK8: 8 weeks after trauma, M3: 3 months after trauma, M6: 6 months after trauma, M12: 12 months after trauma.

## Data Availability

The data utilized in the preparation of this manuscript are publicly available in the National Institute of Mental Health (NIMH) Data Archive (NDA). The dataset identifier for this study is NIMH Data Archive Digital Object Identifier (DOI) 10.15154/zwyn-rb26.

## References

[1] Pitman R. K. Biological studies of post-traumatic stress disorder. Nature Reviews Neuroscience vol. 13 769–787 Preprint at 10.1038/nrn3339 (2012). PMC4951157 23047775

[2] Pacella M. L., Hruska B. & Delahanty D. L. The physical health consequences of PTSD and PTSD symptoms: A meta-analytic review. Journal of Anxiety Disorders vol. 27 33–46 Preprint at 10.1016/j.janxdis.2012.08.004 (2013). 23247200

[3] Schmidt U., Kaltwasser S. F. & Wotjak C. T. Biomarkers in posttraumatic stress disorder: Overview and implications for future research. Disease Markers vol. 35 43–54 Preprint at 10.1155/2013/835876 (2013). 24167348 PMC3774961

[4] Michopoulos V., Norrholm S. D. & Jovanovic T. Diagnostic Biomarkers for Posttraumatic Stress Disorder: Promising Horizons from Translational Neuroscience Research. Biological Psychiatry vol. 78 344–353 Preprint at 10.1016/j.biopsych.2015.01.005 (2015). 25727177 PMC4520791

[5] Al Jowf G. I., Ahmed Z. T., Reijnders R. A., de Nijs L. & Eijssen L. M. T. To Predict, Prevent, and Manage Post-Traumatic Stress Disorder (PTSD): A Review of Pathophysiology, Treatment, and Biomarkers. Int J Mol Sci 24, 5238 (2023). 36982313 10.3390/ijms24065238 PMC10049301

[6] Roeckner A. R., Oliver K. I., Lebois L. A. M., van Rooij S. J. H. & Stevens J. S. Neural contributors to trauma resilience: a review of longitudinal neuroimaging studies. Translational Psychiatry vol. 11 Preprint at 10.1038/s41398-021-01633-y (2021). PMC8492865 34611129

[7] Neria Y. Functional neuroimaging in PTSD: From discovery of underlying mechanisms to addressing diagnostic heterogeneity. American Journal of Psychiatry vol. 178 128–135 Preprint at https://oi.org/10.1176/appi.ajp.2020.20121727 (2021). 33517750 10.1176/appi.ajp.2020.20121727

[8] Carrion V. G., Wong S. S. & Kletter H. Update on Neuroimaging and Cognitive Functioning in Maltreatment-Related Pediatric PTSD: Treatment Implications. J Fam Violence 28, 53–61 (2013).

[9] Chen H. J. Altered resting-state dorsal anterior cingulate cortex functional connectivity in patients with post-traumatic stress disorder. Australian and New Zealand Journal of Psychiatry 53, 68–79 (2019). 30453750 10.1177/0004867418812674

[10] Shaw M. E. Abnormal functional connectivity in posttraumatic stress disorder. Neuroimage 15, 661–674 (2002). 11848709 10.1006/nimg.2001.1024

[11] Jin C. Abnormalities in whole-brain functional connectivity observed in treatment-naive post-traumatic stress disorder patients following an earthquake. Psychol Med 44, 1927–1936 (2014). 24168716 10.1017/S003329171300250X

[12] van Rooij S. J. H. The Role of the Hippocampus in Predicting Future Posttraumatic Stress Disorder Symptoms in Recently Traumatized Civilians. Biol Psychiatry 84, 106–115 (2018). 29110899 10.1016/j.biopsych.2017.09.005 PMC5860925

[13] Stevens J. S. Disrupted amygdala-prefrontal functional connectivity in civilian women with posttraumatic stress disorder. J Psychiatr Res 47, 1469–1478 (2013). 23827769 10.1016/j.jpsychires.2013.05.031 PMC3743923

[14] Breukelaar I. A., Bryant R. A. & Korgaonkar M. S. The functional connectome in posttraumatic stress disorder. Neurobiol Stress 14, (2021). 10.1016/j.ynstr.2021.100321 PMC8065342 33912628

[15] Lebois L. A. M. Large-scale functional brain network architecture changes associated with trauma-related dissociation. American Journal of Psychiatry 178, 165–173 (2021). 32972201 10.1176/appi.ajp.2020.19060647 PMC8030225

[16] Garrett A. Longitudinal changes in brain function associated with symptom improvement in youth with PTSD. J Psychiatr Res 114, 161–169 (2019). 31082658 10.1016/j.jpsychires.2019.04.021 PMC6633919

[17] Malivoire B. L., Girard T. A., Patel R. & Monson C. M. Functional connectivity of hippocampal subregions in PTSD: Relations with symptoms. BMC Psychiatry 18, (2018). 10.1186/s12888-018-1716-9 PMC5952576 29764396

[18] Suo X. Individualized Prediction of PTSD Symptom Severity in Trauma Survivors From Whole-Brain Resting-State Functional Connectivity. Front Behav Neurosci 14, (2020). 10.3389/fnbeh.2020.563152 PMC7779396 33408617

[19] Belleau E. L. Amygdala functional connectivity in the acute aftermath of trauma prospectively predicts severity of posttraumatic stress symptoms: Functional connectivity predicts future PTSD symptoms. Neurobiol Stress 12, (2020). 10.1016/j.ynstr.2020.100217 PMC7231977 32435666

[20] Harnett N. G. Prognostic neuroimaging biomarkers of trauma-related psychopathology: resting-state fMRI shortly after trauma predicts future PTSD and depression symptoms in the AURORA study. Neuropsychopharmacology 46, 1263–1271 (2021). 33479509 10.1038/s41386-020-00946-8 PMC8134491

[21] Schumacher J. Dynamic functional connectivity changes in dementia with Lewy bodies and Alzheimer’s disease. Neuroimage Clin 22, 101812 (2019). 30991620 10.1016/j.nicl.2019.101812 PMC6462776

[22] Rabany L. Dynamic functional connectivity in schizophrenia and autism spectrum disorder: Convergence, divergence and classification. Neuroimage Clin 24, 101966 (2019). 31401405 10.1016/j.nicl.2019.101966 PMC6700449

[23] Sendi M. S. E. Aberrant Dynamic Functional Connectivity of Default Mode Network in Schizophrenia and Links to Symptom Severity. Front Neural Circuits 15, 1–14 (2021). 10.3389/fncir.2021.649417 PMC8013735 33815070

[24] Dini H. Dynamic Functional Connectivity Predicts Treatment Response to Electroconvulsive Therapy in Major Depressive Disorder. Front Hum Neurosci 15, 1–11 (2021). 10.3389/fnhum.2021.689488 PMC8291148 34295231

[25] Sendi M. S. E. Aberrant dynamic functional connectivity of default mode network predicts symptom severity in major depressive disorder. Brain Connect (2021) doi:10.1089/brain.2020.0748. PMC8713570 33514278

[26] Allen E. A. Tracking whole-brain connectivity dynamics in the resting state. Cerebral Cortex 24, 663–676 (2014). 23146964 10.1093/cercor/bhs352 PMC3920766

[27] Preti M. G., Bolton T. A. & Van De Ville D. The dynamic functional connectome: State-of-the-art and perspectives. Neuroimage 160, 41–54 (2017). 28034766 10.1016/j.neuroimage.2016.12.061

[28] Calhoun V. D., Miller R., Pearlson G. & Adali T. The Chronnectome: Time-Varying Connectivity Networks as the Next Frontier in fMRI Data Discovery. Neuron vol. 84 262–274 Preprint at 10.1016/j.neuron.2014.10.015 (2014). PMC4372723 25374354

[29] Fu Z. Dynamic functional network connectivity associated with post-traumatic stress symptoms in COVID-19 survivors. Neurobiol Stress 15, (2021). 10.1016/j.ynstr.2021.100377 PMC8339567 34377750

[30] Dai Y. Altered dynamic functional connectivity associates with post-traumatic stress disorder. Brain Imaging Behav (2023) doi:10.1007/s11682-023-00760-y. 36826627

[31] Wen Z., Seo J., Pace-Schott E. F. & Milad M. R. Abnormal dynamic functional connectivity during fear extinction learning in PTSD and anxiety disorders. Mol Psychiatry 27, 2216–2224 (2022). 35145227 10.1038/s41380-022-01462-5 PMC9126814

[32] Fu S. Altered local and large-scale dynamic functional connectivity variability in posttraumatic stress disorder: A resting-state fMRI study. Front Psychiatry 10, (2019). 10.3389/fpsyt.2019.00234 PMC6474202 31031661

[33] Dobie D. J. Posttraumatic Stress Disorder in Female Veterans Association With Self-reported Health Problems and Functional Impairment. Arch Intern Med 164, 394–400 (2004). 14980990 10.1001/archinte.164.4.394

[34] Golin C. E. Post-traumatic stress disorder symptoms and mental health over time among low-income women at increased risk of HIV in the U.S. J Health Care Poor Underserved 27, 891–910 (2016). 27180715 10.1353/hpu.2016.0093 PMC4970215

[35] Rees S. J. A high-risk group of pregnant women with elevated levels of conflict-related trauma, intimate partner violence, symptoms of depression and other forms of mental distress in post-conflict Timor-Leste. Transl Psychiatry 6, (2016). 10.1038/tp.2015.212 PMC4872420 26836413

[36] Rayburn N. R. Trauma, depression, coping, and mental health service seeking among impoverished women. J Consult Clin Psychol 73, 667–677 (2005). 16173854 10.1037/0022-006X.73.4.667

[37] Webb E. K. Neural impact of neighborhood socioeconomic disadvantage in traumatically injured adults. Neurobiol Stress 15, (2021). 10.1016/j.ynstr.2021.100385 PMC8390770 34471656

[38] McLean S. A. The AURORA Study: a longitudinal, multimodal library of brain biology and function after traumatic stress exposure. Mol Psychiatry 25, 283–296 (2020). 31745239 10.1038/s41380-019-0581-3 PMC6981025

[39] Bovin M. J. Psychometric properties of the PTSD checklist for diagnostic and statistical manual of mental disorders-fifth edition (PCL-5) in veterans. Psychol Assess 28, 1379–1391 (2016). 26653052 10.1037/pas0000254

[40] Du Y., Fu Z. & Calhoun V. D. Classification and prediction of brain disorders using functional connectivity: Promising but challenging. Frontiers in Neuroscience vol. 12 Preprint at 10.3389/fnins.2018.00525 (2018). PMC6088208 30127711

[41] Jin C. Dynamic brain connectivity is a better predictor of PTSD than static connectivity. Hum Brain Mapp 38, 4479–4496 (2017). 28603919 10.1002/hbm.23676 PMC6866943

[42] Tozlu C., Jamison K., Gauthier S. A. & Kuceyeski A. Dynamic Functional Connectivity Better Predicts Disability Than Structural and Static Functional Connectivity in People With Multiple Sclerosis. Front Neurosci 15, (2021). 10.3389/fnins.2021.763966 PMC8710545 34966255

[43] Sendi M. S. E. Alzheimer’s Disease Projection From Normal to Mild Dementia Reflected in Functional Network Connectivity: A Longitudinal Study. Front Neural Circuits 14, (2021). 10.3389/fncir.2020.593263 PMC7859281 33551754

[44] MacGregor A. J., Joseph A. R., Walker G. J. & Dougherty A. L. Co-occurrence of hearing loss and posttraumatic stress disorder among injured military personnel: A retrospective study. BMC Public Health 20, (2020). 10.1186/s12889-020-08999-6 PMC7341578 32641028

[45] Mueller-Pfeiffer C. Atypical visual processing in posttraumatic stress disorder. Neuroimage Clin 3, 531–538 (2013). 24371791 10.1016/j.nicl.2013.08.009 PMC3871398

[46] Rangaprakash D., Dretsch M. N., Katz J. S., Denney T. S. & Deshpande G. Dynamics of segregation and integration in directional brain networks: Illustration in soldiers with PTSD and neurotrauma. Front Neurosci 13, (2019). 10.3389/fnins.2019.00803 PMC6716456 31507353

[47] Lobo I. Hidden wounds of violence: Abnormal motor oscillatory brain activity is related to posttraumatic stress symptoms. Neuroimage 224, (2021). 10.1016/j.neuroimage.2020.117404 32971264

[48] Harnett N. G. Structural covariance of the ventral visual stream predicts posttraumatic intrusion and nightmare symptoms: a multivariate data fusion analysis. Transl Psychiatry 12, (2022). 10.1038/s41398-022-02085-8 PMC9360028 35941117

[49] Harnett N. G. Acute Posttraumatic Symptoms Are Associated With Multimodal Neuroimaging Structural Covariance Patterns: A Possible Role for the Neural Substrates of Visual Processing in Posttraumatic Stress Disorder. Biol Psychiatry Cogn Neurosci Neuroimaging 7, 129–138 (2022). 33012681 10.1016/j.bpsc.2020.07.019 PMC7954466

[50] Zhang Y. Intranetwork and internetwork functional connectivity alterations in post-traumatic stress disorder. J Affect Disord 187, 114–121 (2015). 26331685 10.1016/j.jad.2015.08.043

[51] Shang J. Alterations in low-level perceptual networks related to clinical severity in PTSD after an earthquake: A resting-state fMRI study. PLoS One 9, (2014). 10.1371/journal.pone.0096834 PMC4019529 24823717

[52] Avrahami D. Visual art therapy’s unique contribution in the treatment of post-traumatic stress disorders. Journal of Trauma and Dissociation 6, 5–38 (2005). 10.1300/j229v06n04_02 16537321

[53] Blithikioti C. The cerebellum and psychological trauma: A systematic review of neuroimaging studies. Neurobiology of Stress vol. 17 Preprint at 10.1016/j.ynstr.2022.100429 (2022). PMC8801754 35146077

[54] Baldaçara L. Reduced cerebellar left hemisphere and vermal volume in adults with PTSD from a community sample. J Psychiatr Res 45, 1627–1633 (2011). 21824628 10.1016/j.jpsychires.2011.07.013

[55] Lebois L. A. M. Persistent Dissociation and Its Neural Correlates in Predicting Outcomes After Trauma Exposure. American Journal of Psychiatry 179, 661–672 (2022). 35730162 10.1176/appi.ajp.21090911 PMC9444876

[56] Fonkoue I. T., Michopoulos V. & Park J. Sex differences in post-traumatic stress disorder risk: autonomic control and inflammation. Clinical Autonomic Research vol. 30 409–421 Preprint at 10.1007/s10286-020-00729-7 (2020). 33021709 PMC7598146

[57] Nzimande N. P. Sex differences in the experience of COVID-19 post-traumatic stress symptoms by adults in South Africa. BMC Psychiatry 22, (2022). 10.1186/s12888-022-03883-6 PMC8977834 35379197

[58] Olff M. Sex and gender differences in post-traumatic stress disorder: an update. Eur J Psychotraumatol 8, (2017).

[59] Olff M. Sex and gender differences in post-traumatic stress disorder: an update. Eur J Psychotraumatol 8, (2017).

[60] Olff M. Sex and gender differences in post-traumatic stress disorder: an update. Eur J Psychotraumatol 8, (2017).

[61] Duncan L. E. Largest GWAS of PTSD (N=20 070) yields genetic overlap with schizophrenia and sex differences in heritability. Mol Psychiatry 23, 666–673 (2018). 28439101 10.1038/mp.2017.77 PMC5696105

[62] Nievergelt C. M. International meta-analysis of PTSD genome-wide association studies identifies sex- and ancestry-specific genetic risk loci. Nat Commun 10, (2019). 10.1038/s41467-019-12576-w PMC6783435 31594949

[63] Rowland G. E. Prior Sexual Trauma Exposure Impacts Posttraumatic Dysfunction and Neural Circuitry Following a Recent Traumatic Event in the AURORA Study. Biological Psychiatry Global Open Science 3, 705–715 (2023). 37881578 10.1016/j.bpsgos.2023.02.004 PMC10593890

[64] Harnett N. G. Structural inequities contribute to racial/ethnic differences in neurophysiological tone, but not threat reactivity, after trauma exposure. Mol Psychiatry (2023) doi:10.1038/s41380-023-01971-x. PMC10615735 36725899

[65] Sendi M. S. E. The link between static and dynamic brain functional network connectivity and genetic risk of Alzheimer’s disease. Neuroimage Clin 37, (2023). 10.1016/j.nicl.2023.103363 PMC9999198 36871405

[66] Du Y. NeuroMark: An automated and adaptive ICA based pipeline to identify reproducible fMRI markers of brain disorders. Neuroimage Clin 28, 102375 (2020). 32961402 10.1016/j.nicl.2020.102375 PMC7509081

[67] Sendi M. S. E. Alzheimer’s Disease Projection From Normal to Mild Dementia Reflected in Functional Network Connectivity: A Longitudinal Study. Front Neural Circuits 14, (2021). 10.3389/fncir.2020.593263 PMC7859281 33551754

[68] Calhoun V. D., Liu J. & Adali T. A review of group ICA for fMRI data and ICA for joint inference of imaging, genetic, and ERP data. Neuroimage 45, (2009). 10.1016/j.neuroimage.2008.10.057 PMC2651152 19059344

[69] Yaesoubi M., Adalı T. & Calhoun V. D. A window-less approach for capturing time-varying connectivity in fMRI data reveals the presence of states with variable rates of change. Hum Brain Mapp 39, 1626–1636 (2018). 29315982 10.1002/hbm.23939 PMC5847478

[70] Jafri M. J., Pearlson G. D., Stevens M. & Calhoun V. D. A method for functional network connectivity among spatially independent resting-state components in schizophrenia. Neuroimage 39, 1666–1681 (2008). 18082428 10.1016/j.neuroimage.2007.11.001 PMC3164840

[71] Allen E. A. Tracking whole-brain connectivity dynamics in the resting state. Cerebral Cortex 24, 663–676 (2014). 23146964 10.1093/cercor/bhs352 PMC3920766

